# Glucose phosphate isomerase deficiency demasked by whole-genome sequencing: a case report

**DOI:** 10.1186/s13256-024-04466-7

**Published:** 2024-03-28

**Authors:** Sissel Holme, Richard van Wijk, Andreas Ørslev Rasmussen, Jesper Petersen, Andreas Glenthøj

**Affiliations:** 1 Danish Red Blood Cell Center, Department of Hematology, Copenhagen University Hospital – Rigshospitalet, Copenhagen, Denmark; 2 Central Diagnostic Laboratory, University Medical Center Utrecht, Utrecht University, Utrecht, The Netherlands; 3 Center for Genomic Medicine, Rigshospitalet, Copenhagen University Hospital - Rigshospitalet, Copenhagen, Denmark; 4 Department of Clinical Medicine, University of Copenhagen, Copenhagen, Denmark

**Keywords:** Glucose-6-phosphate isomerase, Hemolytic anemia, Glycolysis, RBC enzymes, Hereditary anemia

## Abstract

**Background:**

Glucose-6-phosphate isomerase deficiency is a rare genetic disorder causing hereditary nonspherocytic hemolytic anemia. It is the second most common glycolytic enzymopathy in red blood cells. About 90 cases are reported worldwide, with symptoms including chronic hemolytic anemia, jaundice, splenomegaly, gallstones, cholecystitis, and in severe cases, neurological impairments, hydrops fetalis, and neonatal death.

**Case presentation:**

This paper details the case of the first Danish patient diagnosed with glucose-6-phosphate isomerase deficiency. The patient, a 27-year-old white female, suffered from lifelong anemia of unknown origin for decades. Diagnosis was established through whole-genome sequencing, which identified two *GPI* missense variants: the previously documented variant p.(Thr224Met) and a newly discovered variant p.(Tyr341Cys). The pathogenicity of these variants was verified enzymatically.

**Conclusions:**

Whole-genome sequencing stands as a potent tool for identifying hereditary anemias, ensuring optimal management strategies.

**Supplementary Information:**

The online version contains supplementary material available at 10.1186/s13256-024-04466-7.

## Introduction

Glucose-6-phosphate isomerase (GPI) deficiency is a rare autosomal recessive disorder caused by homozygous or compound heterozygous variations in the *GPI* gene [1]. It is the second most common glycolytic enzymopathy in red blood cells (RBCs) after pyruvate kinase deficiency. GPI plays a crucial role in glycolysis by converting glucose-6-phosphate into fructose-6-phosphate. The resulting imbalance disrupts RBC metabolism and leads to hemolysis [2].

Most cases are diagnosed in neonatal and early childhood, causing hereditary nonspherocytic hemolytic anemia with chronic hemolysis and possible acute crises due to infections [1].

Symptoms are mild-to-severe anemia with fatigue, tachycardia, dyspnea, and pallor. Other symptoms include jaundice, splenomegaly, gallstones, and cholecystitis, and in a few severe cases, it has been shown to cause neurological deficits, hydrops fetalis, and neonatal death [1, 3].

The first report of the disease was described in 1968 by Baughan *et al.* [4], and since then about 90 patients have been diagnosed from a variety of ethnic groups and populations throughout the world, and over 100 different variations associated with GPI deficiency have been identified [1].

Diagnosis involves measuring GPI activity in RBCs and genetic testing with detection and confirmation of pathogenic variants of *GPI* [5, 6].

Treatment is primarily supportive, involving transfusions and chelation therapy, and often necessitates splenectomy [2].

In this case report, we describe the presentation and diagnosis of the first patient in Denmark diagnosed with GPI deficiency.

## Case presentation

The patient is a 27-year-old woman of Venezuelan and Italian descent who was referred to our institution in 2018 owing to lifelong macrocytic hemolytic anemia.

As a newborn, she had jaundice and dilated bile ducts, leading to a diagnosis of chronic hemolytic anemia. Her gallbladder was subsequently removed. The patient was previously tested in other countries without explanation for the etiology and was initially misdiagnosed as having hereditary spherocytosis.

She experienced worsening symptoms during periods of stress, menstruation, and infections, including increased fatigue, yellowing of the eyes, dark urine, difficulty breathing, and chest pain. Episodes of jaundice decreased with a shift to a more plant-based diet, and they no longer occur during menstruation.

There is no family history of chronic hemolysis, but her grandmother had gallstones, and her grandfather was diagnosed with chronic myeloid leukemia.

## Clinical findings

Physical examination revealed no pathological findings or neurological impairment. Blood tests showed moderate hemolytic anemia with macrocytosis, reticulocytosis, hyperbilirubinemia, and decreased haptoglobin, consistent with hemolysis (Table 1).

**Table 1:** Patient’s hematological and biochemical parameters, compared against standard reference ranges

Parameter	Value	Reference value
Hgb (g/dL)	10.3	11.8–15.3
MCV (fL)	113	82–98
MCHC (g/dL)	30.29	31.7–35.8
Reticulocytes (× 10^9^/L)	256	25–99
Reticulocytes (%)	9.0	0.5–2.2
Haptoglobin (g/L)	0.18	0.35–1.85
Bilirubin (mg/dL	2.63	0.29–1.46
Lactate dehydrogenase (U/L)	187	105–205
Platelets (× 10^9^/L)	400	145–390
RDW (%)	13%	< 16

Hemoglobin (Hgb), mean corpuscular volume (MCV), mean corpuscular hemoglobin concentration (MCHC), absolute reticulocyte count (Reticulocytes), haptoglobin, bilirubin, lactate dehydrogenase, platelet count (platelets), and red cell distribution width (RDW)

**Fig. 1:**
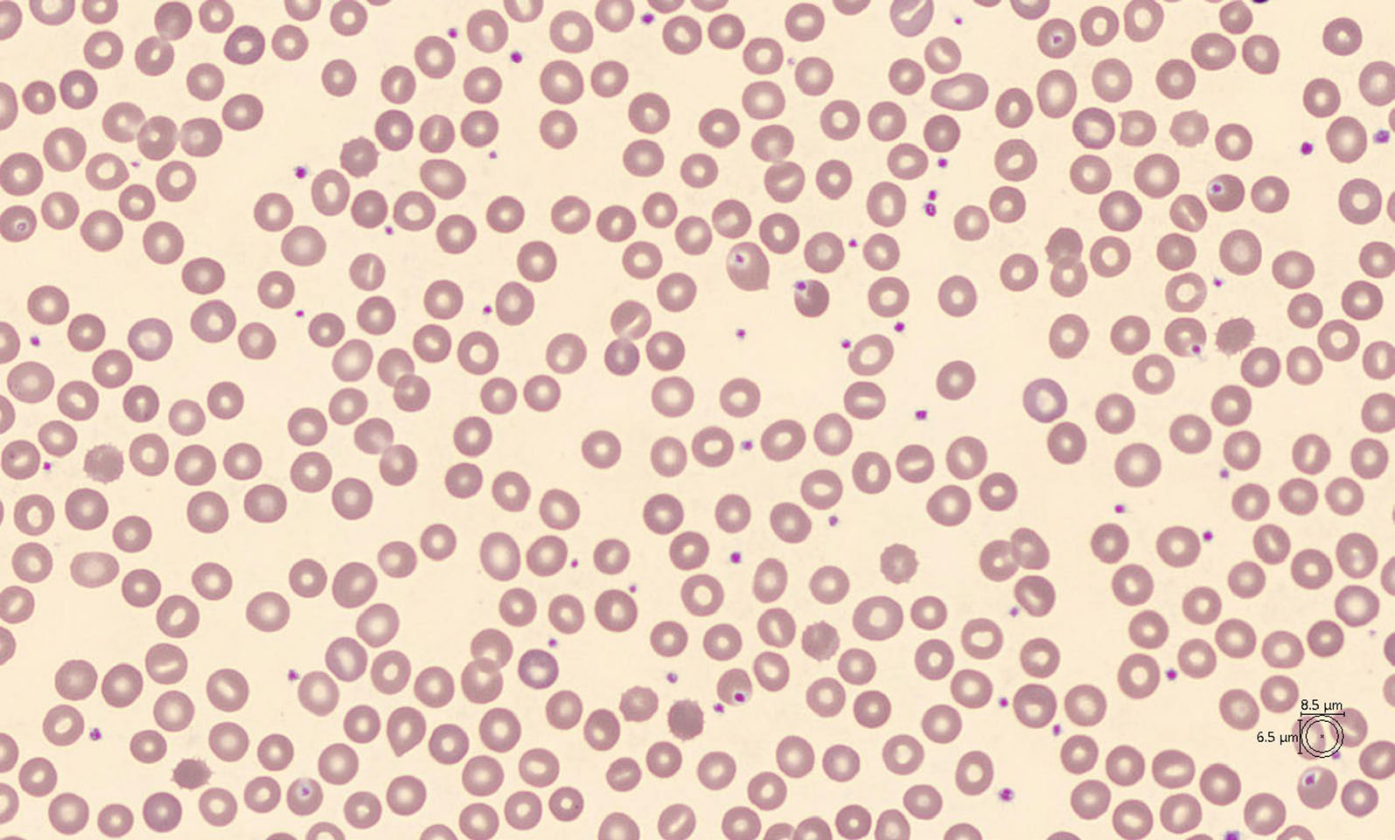
Peripheral blood smear. Peripheral blood smear using a Sysmex DI-60 digital imaging analyzer and CellaVision 7.0 with Advance Red red blood cell application. Mild stomatocytosis is noted

Various tests, including direct anti-globulin test, osmotic gradient ektacytometry [7, 8], *PKLR* sequencing, blood smear, and hemoglobin electrophoresis [9], ruled out other diagnoses such as hereditary spherocytosis, pyruvate kinase deficiency, autoimmune hemolytic diseases, glucose-6-phosphate dehydrogenase deficiency, thalassemia, and sickle-cell disease. Peripheral blood smear was unremarkable aside from mild stomatocytosis (Fig. 1).

**Fig. 2:**
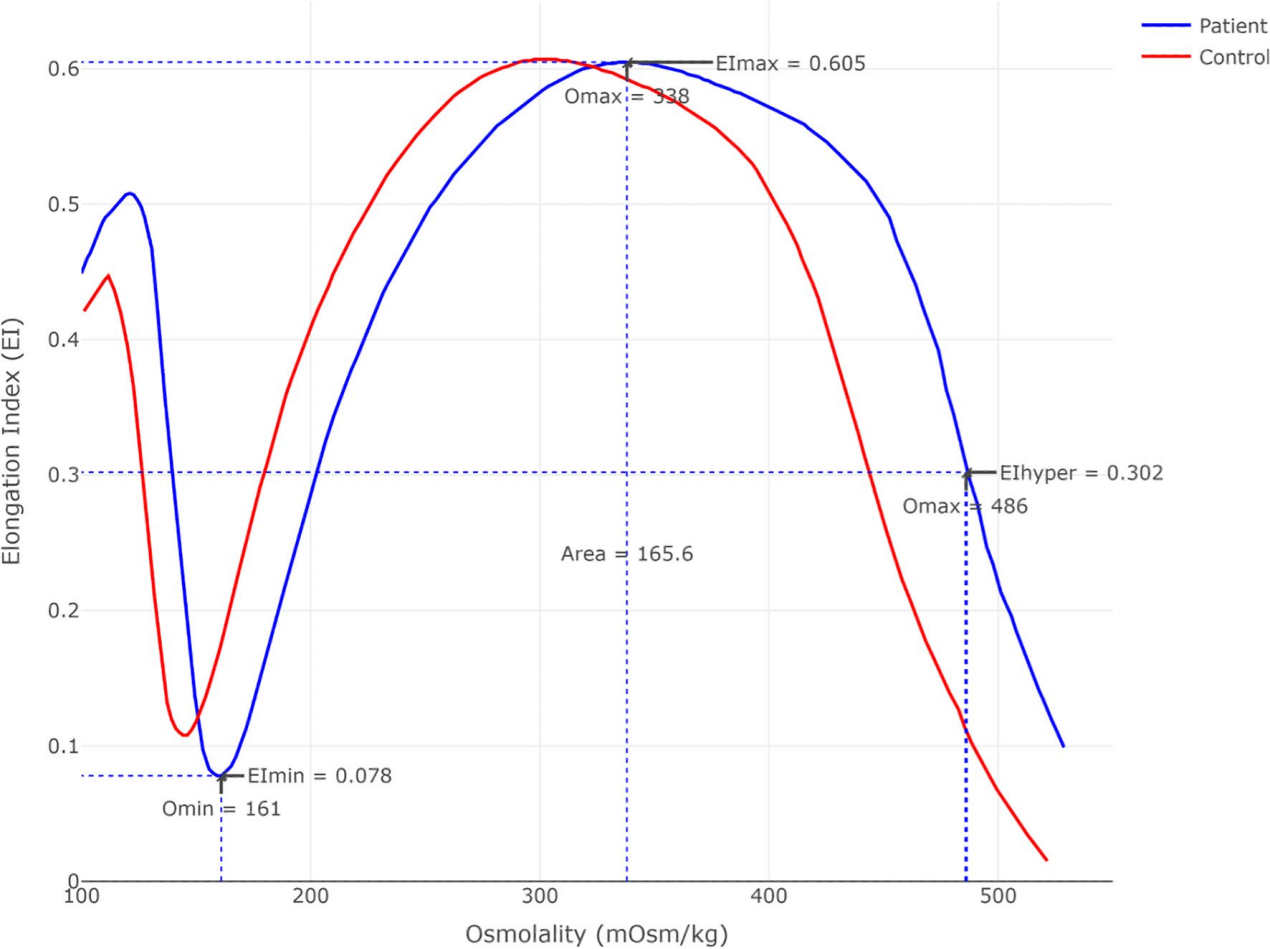
Ektacytometry. Osmotic gradient ektacytometry was performed on ethylenediaminetetraacetic acid–blood on a LoRRca ektacytometer (RR Mechatronics, Zwaag, Netherlands) within 48 h of venipuncture according to manufacturer’s instructions. The patient’s ektacytometry curve was right shifted (O_min_ = 161 mOsm/kg, O_max_ = 338 mOsm/kg) but with normal maximal deformability (EI_max_ = 0.605)

Similar to a previous report, the patient's ektacytometry (Fig. 2) curve was right-shifted and did not support a diagnosis of hereditary spherocytosis [10].

Whole-genome sequencing (WGS) was performed at the Danish National Genome Center (https://eng.ngc.dk) according to their standard procedures, and variants were subsequently filtered using a custom anemia in silico gene panel (Additional file 1). WGS revealed two likely disease-causing variants in the *GPI* gene (NM_000175.5, NP_000166.2). The first detected missense variant (c.671C > T, p.(Thr224Met)) has an overall allele frequency in the background population of 0.0039% (gnomAD v3.1.2) and alters a moderately to highly conserved amino acid.

In silico analysis (REVEL [11]) indicated an increased likelihood that the variant is pathogenic (score: 0.852), and it has been documented in patients with GPI deficiency in both homozygous (Kanno *et al.*, 1996 [12]) and compound heterozygous forms (Xu *et al.*, 1994 [13]). The variant has also been shown to co-segregate with the disease in a family [12]. The variant was classified as pathogenic according to American College of Medical Genetics and Genomics (ACMG) guidelines [14].

The second detected missense variant (c.1022A > G, p.(Tyr341Cys)) in *GPI* has an overall allele frequency of 0.0026% (gnomAD v3.1.2) and modifies a highly conserved amino acid. Despite its absence in prior literature or functional studies, in silico analysis (REVEL) suggested a high probability of pathogenicity (score: 0.987), leading to its classification as likely pathogenic according to ACMG and ClinGen guidelines [15].

Enzymatic assays showed a GPI activity of 7 U/g Hb (normal range 32–72 U/g Hb) and elevated hexokinase (HK) activity of 3.4 U/g Hb (normal range 0.8–1.5 U/g Hb). The strongly decreased GPI activity supports the diagnosis of GPI deficiency. The absence of available relatives for testing impeded our ability to clearly discern the distinct impacts of the two *GPI* variants. Ideally, we would have preferred this to comprehensively authenticate the novel Tyr431Cys variant. The HK activity is measured as a reference to evaluate mean red cell age.

### Treatment and follow-up

Currently, the patient’s therapeutic intervention consists only of folic acid. Although her hemoglobin has been steadily low, blood transfusions or splenectomy have not been necessary. Activators of the glycolytic pathway such as mitapivat, etovapivat, and AG946 are either approved or under clinical investigation for other hereditary anemias. However, these activators target pyruvate kinase (PK), which acts downstream of GPI in the glycolytic pathway. Presuming that PK activation would have an impact, this could potentially reduce hemolysis by enhancing the availability of adenosine triphosphate (ATP) in GPI-deficient RBCs. Nevertheless, PK activators are also known to decrease the level of the glycolytic intermediate 2,3-Diphosphoglycerate (2,3-DPG). This in turn elevates the oxygen affinity of hemoglobin, which subsequently diminishes oxygen delivery to tissues.

## Discussion

GPI deficiency is the second most common glycolytic enzymopathy in RBCs after PK deficiency, but its exact frequency is unknown, and it is likely underdiagnosed owing to the lack of awareness and availability of testing. Diagnosis of GPI deficiency can be challenging, and genetic testing has become an important tool in the diagnostic process. Enzymatic assays are scarcely available and most often require fresh blood shipment to highly specialized laboratories. Contrarily, next-generation sequencing (NGS)-based methods such as WGS are becoming increasingly available for hemolytic anemia owing to a decrease in cost and do not require special handling.

Lack of knowledge about rare anemias as well as cumbersome testing can delay or prevent diagnosis of rare anemias. Furthermore, the clinical presentation of chronic hemolytic anemia can be similar, requiring multiple tests to exclude other diagnoses [6].

This patient exhibited consistently low hemoglobin, moderate hemolysis, intermittent jaundice, and hyperbilirubinemia, but no splenomegaly. It remains uncertain whether a splenectomy may become necessary in the future. Splenectomy has shown to reduce hemolysis and dependence on transfusion [16].

The implementation and advancement of next-generation sequencing have improved routine diagnostic workup of hereditary anemias. It can certainly aid in diagnosing and thereby correctly managing GPI deficiency as well as other ultrarare hereditary anemias. However, it is important to note that genetic testing may not always identify the specific variant responsible for GPI deficiency, and for novel variants, estimation of pathogenicity is usually based on prediction. Therefore, additional testing, such as enzymatic assays, in this case, remains necessary [17].

No curative or targeted treatments exist for this disorder. Regular blood transfusions may be necessary for patients, including monitoring for iron overload. Promising new treatment options, including activators of the glycolytic pathway, are currently being clinically investigated for numerous other hereditary anemias, but to date, no clinical studies have targeted GPI deficiency. Establishing diagnosis and mapping patients with rare anemias is pivotal for research into these debilitating diseases and a prerequisite for clinical trials on novel treatment options.

## Conclusion

This case report underscores the significance of diagnosing and managing GPI deficiency. The complexity and rarity of this condition often lead to misdiagnosis, emphasizing the value of advanced diagnostic tools such as WGS. While supportive care is the current approach, potential treatments targeting the glycolytic pathway offer hope. Establishing accurate diagnosis and understanding of rare anemias remains crucial for advancing research and exploring innovative therapies.

### Supplementary Information

**Additional file 1.** Custom in silico gene panel used for patients suspected of hereditary anemia.

## Data Availability

The data that support the findings of this study are available from the corresponding author upon reasonable request.
