# Supplementary material for: Glucose phosphate isomerase deficiency demasked by whole-genome sequencing: a case report
Source: J Med Case Rep. 2024 Mar 28;18:130. doi: 10.1186/s13256-024-04466-7 (PMC10976829; doi:10.1186/s13256-024-04466-7)
Supplement: Supplementary file 1 — Additional file 1. Custom in silico gene panel used for patients suspected of hereditary anemia. [file 13256_2024_4466_MOESM1_ESM.docx]

Supplementary data 1

**Hematological *in silico* gene panels v3**

**Membranopathy**

ADD1 (NM_014189.3), ADD2 (NM_001617.3), AK1 (NM_000476.2), ANK1 (NM_001142446.1), APOB (NM_000384.2), DMTN (NM_001978), EPB41 (NM_001166005.1), EPB42 (NM_000119.2), KCNN4 (NM_002250.2), MTTP (NM_001300785.1), PIEZO1 (NM_001142864.3), RhAG (NM_000324.2), SLC2A1 (NM_006516.2), SLC4A1 (NM_000342.3), SPTA1 (NM_003126.2), SPTB (NM_001024858.2), STOM (NM_004099.5), TMOD1 (NM_003275.3), TPM3 (NM_153649.3), XK (NM_021083.3).

**Red Cell Enzyme**

ALDOA (NM_000034.3), BPGM (NM_199186.2), CYB5A (NM_148923.3), CYB5R1 (NM_016243.2), CYB5R2 (NM_001302826.1), CYB5R3 (NM_001129819.2), CYB5R4 (NM_016230.3), CYB5RL (NM_001031672.2), ENO1 (NM_001428.3), G6PD (NM_000402.4), GAPDH (NM_002046.5), GCLC (NM_001498.3), GPI (NM_001289789.1), GPX1 (NM_000581.3), GSR (NM_000637.3), GSS (NM_000178.3), HK1 (NM_033496.2), HK2 (NM_000189.4), NT5C3A (NM_001002010.2), PFKM (NM_001166686.1), PGAM1 (NM_002629.2), PGD (NM_002631.3), PGK1 (NM_000291.3), PGM1 (NM_001172818.1), PKLR (NM_000298.5), TPI1 (NM_001159287.1).

**Hemoglobinopathies**

AHSP (NM_016633.3), ATRX (NM_000489.4), HBA1 (NM_000558.4), HBA2 (NM_000517.4), HBB (NM_000518.4), HBD (NM_000519.3), HBE1 (NM_005330.3), HBG1 (NM_000559.2), HBG2 (NM_000184.2), HBM (NM_001003938.3), HBQ1 (NM_005331.4), HBZ (NM_005332.2), SUPT5H (NM_001111020.3).

**Congenital Dyserythropeoietic Anaemia**

CDAN1 (NM_138477.2), C15ORF41 (NM_001130010.2), COX4I2 (NM_032609.2), GATA1 (NM_002049.3), GATA2 (NM_032638.4), KIF23 (NM_138555.3), KLF1 (NM_006563.4), LPIN2 (NM_014646.2), SEC23B (NM_032985.4), TAL1 (NM_003189.5), RACGAP1 (NM_013277).

**Megaloblastic Anaemia**

AMN (NM_030943.3), CUBN (NM_001081.3), DHFR (NM_000791), FTCD (NM_206965.1), GIF (NM_005142.2), COX1 YP_003024028.1), MTR (NM_000254.2), MTRR (NM_024010.2), SLC19A2 (NM_006996.2), UMPS (NM_000373.3), TCN2 (NM_000355.4).

**Congenital Erythrocytosis**

BHLHE41 (NM_030762.2), BPGM (NM_199186.2), EGLN1 (NM_022051.2), EGLN2 (NM_080732.3), EGLN3 (NM_022073.3), EPAS1 (NM_001430.4), EPO (NM_000799.2), EPOR (NM_000121.3), GFI1B (NM_004188.6), HBA1 (NM_000558.4), HBA2 (NM_000517.4), HBB (NM_000518.4), HIF1A (NM_001243084.1), HIF1AN (NM_017902.2), HIF3A (NM_152795.3), JAK2 (NM_004972.3), KDM6A (NM_001291415.1), OS9 (NM_006812.3), SH2B3 (NM_005475.2), VHL (NM_000551.3), ZNF197 (NM_006991.4), PIEZO1 (NM_001142864.3).

**Diamond Blackfan Anaemia**

RPL5 (NM_000969.3), RPL9 (NM_000661.4), RPS10 (NM_001203245.2), RPL11 (NM_000975.3), RPL15 (NM_002948.3), RPL19 (NM_000981.3), RPL26 (NM_001315530.1), RPL27 (NM_000988.3), RPL35A (NM_001316311.1), RPS7 (NM_001011.3), RPS19 (NM_001022.3), RPS24 (NM_001142285.1), RPS26 (NM_001029.3), RPS29 (NM_001032.4), ADA2 (NM_001282225.2), RPL31 (NM_000993.5), TSR2 (NM_058163.3), RPS27 (NM_001030.6), RPL35 (NM_007209.4), RPS17 (NM_001021.6), RPS15A (NM_001019.5), HEATR3 (NM_182922.4), RPS28 (NM_001031.5), RPL18 (NM_000979.4), TSR2 (NM_058163.3).

**Bone Marrow Failure**

ACD (NM_001082486.1), CTC1 (NM_025099.5), DKC1 (NM_001363.4), HOXA11 (NM_005523.5), LIG4 (NM_002312.3), MASTL (NM_001320757.1), MPL (NM_005373.2), NHP2 (NM_017838.3), NOP10 (NM_018648.3), NT5C3A (NM_001002010.2), PALB2 (NM_024675.3), PARN (NM_002582.3), RAD51C (NM_058216.2), RMRP NR_003051.3), RTEL1 (NM_001283009.1), SBDS (NM_016038.2), SMARCAL1 (NM_014140.3), SRP72 (NM_006947.3), TERC NR_001566.1), TERT (NM_198253.2), TINF2 (NM_001099274.1), WRAP53 (NM_018081.2), XRCC2 (NM_005431.1).

**Porphyria**

ALAD (NM_000031.5), ALAS2 (NM_000032.4), CPOX (NM_000097.5), FECH (NM_001012515.2), GATA1 (NM_002049.3), HMBS (NM_000190.3), PPOX (NM_001122764.1), UROD (NM_000374.4), UROS (NM_000375.2).

**Neutropenia**

ACKR1 (NM_001122951.2), CECR1 (NM_001282225.1), CXCR4 (NM_001348056.1), ELANE (NM_001972.3), G6PC3 (NM_138387.3), GFI1 (NM_005263.3), HAX1 (NM_006118.3), SLC37A4 (NM_001164278.1), SMARCAL1 (NM_014140.3), TAZ (NM_000116.4), USB1 (NM_024598.3), WAS (NM_000377.2).

**Sideroblastic Anaemia**

ABCB6 (NM_005689.2), ABCB7 (NM_004299.4), ALAS1 (NM_000688.5), ALAS2 (NM_000032.4), GLRX5 (NM_016417.2), PUS1 (NM_001002020.2), SF3B1 (NM_012433.3), SLC19A2 (NM_006996.2), SLC25A38 (NM_017875.2), YARS2 (NM_001040436.2), HSPA9 (NM_004134.7), TRNT1 (NM_182916.3), LARS2 (NM_015340.4), NDUFB11 (NM_001135998.3).

**Iron Regulation**

BMP4 (NM_001202.5), BMP6 (NM_001718), CP (NM_000096.3), FTH1 (NM_002032.2), FTL (NM_000146.3), HAMP (NM_021175), HFE (NM_000410.3), HFE2 (NM_213653.3), SLC11A2 (NM_001174125.1), SLC40A1 (NM_014585), SMAD4 (NM_005359), SMAD6 (NM_005585.4), SMAD7 (NM_005904.3), TF (NM_001063.3), TFR2 (NM_003227.3), TFRC (NM_003234.3), TMPRSS6 (NM_001289000.1), PIGA (NM_002641), NEO1 (NM_002499.4).

**HLH**

PRF1 (NM_001083116.1), STX11 (NM_003764.3), STXBP2 (NM_001272034.1), UNC13D (NM_199242.2).

**Lymphedema**

CCBE1 (NM_133459.3), FAT4 (NM_001291303.1), GATA2 (NM_032638.4), PIEZO1 (NM_001142864.3).

**Beyond the panels (single genes)**

HP (NM_005143.3), SERPINA1 (NM_000295.4), UGT1A1 (NM_000463.2), CD59 (NM_000611.6), STEAP3 (NM_182915.3), ABCG5 (NM_022436.3), ABCG8 (NM_022437.3).
